# Publisher Correction: Encryption and steganography of synthetic gene circuits

**DOI:** 10.1038/s41467-018-07844-0

**Published:** 2019-01-08

**Authors:** Oliver Purcell, Jerry Wang, Piro Siuti, Timothy K. Lu

**Affiliations:** 1 Synthetic Biology Center, Massachusetts Institute of Technology, 500 Technology Square, Cambridge, MA 02139 USA; 2 Department of Biological Engineering, Massachusetts Institute of Technology, 77 Massachusetts Avenue, Cambridge, MA 02139 USA; 3 Research Laboratory of Electronics, Department of Electrical Engineering and Computer Science, Massachusetts Institute of Technology, 77 Massachusetts Avenue, Cambridge, MA 02139 USA

Correction to: *Nature Communications* 10.1038/s41467-018-07144-7; published online 22 November 2018

The original version of this Article contained an error in Fig. 4. In the lower part of the three gene circuit diagrams in panel b, the flat-headed arrow linking lambdaCI to the tetR promoter incorrectly pointed to the tetR gene body. This has now been corrected in the HTML and PDF versions of this Article.

