# Supplementary material for: Encryption and steganography of synthetic gene circuits
Source: Nat Commun. 2018 Nov 22;9:4942. doi: 10.1038/s41467-018-07144-7 (PMC6250736; doi:10.1038/s41467-018-07144-7)
Supplement: Supplementary file 1 — Supplementary Information [file 41467_2018_7144_MOESM1_ESM.pdf]

Supplementary information: Encryption and Steganography of Synthetic Gene Circuits.

Purcell et al.

Supplementary Figure 1

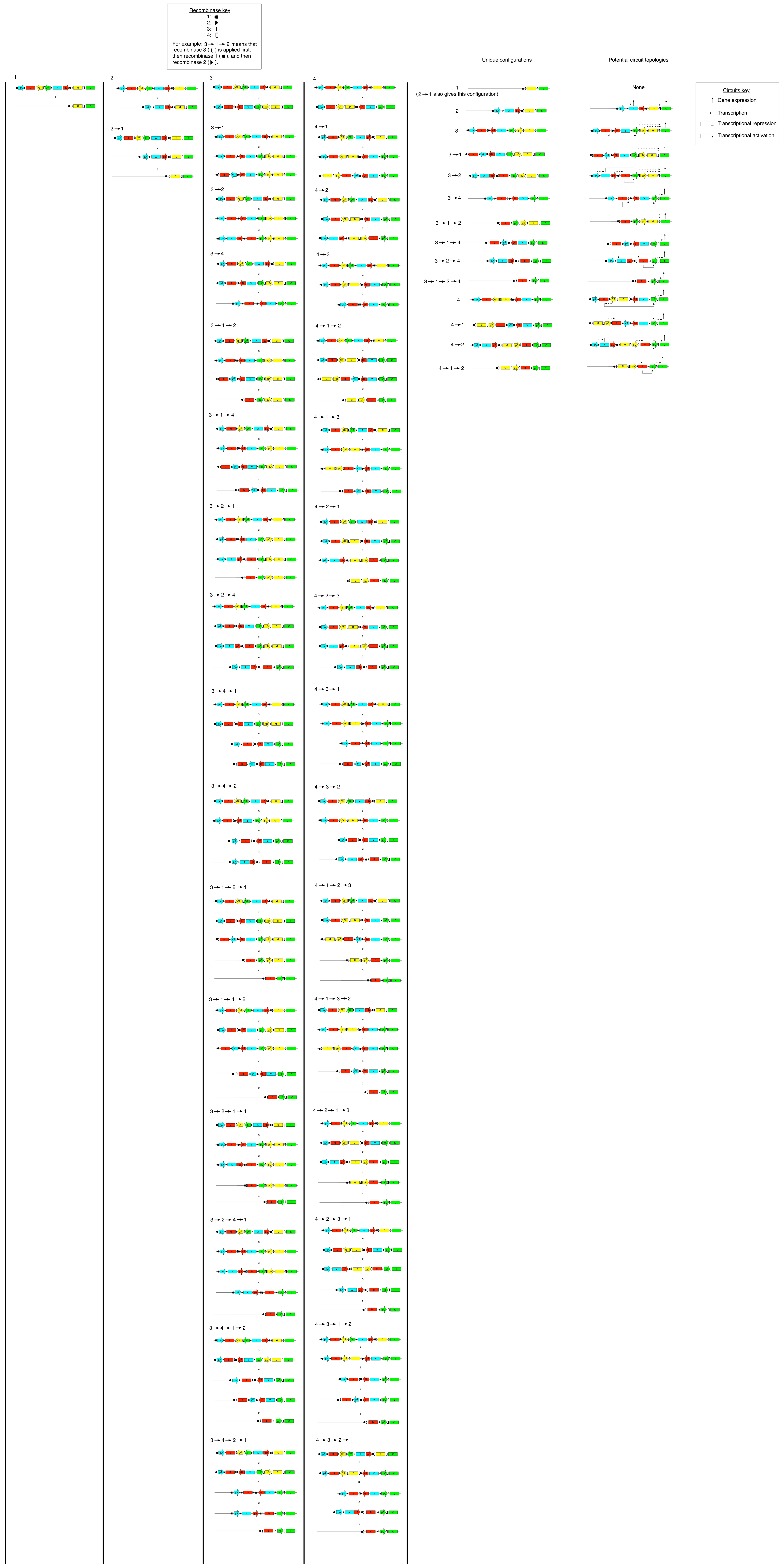

Supplementary Figure 1. All possible decryption routes of the genetic AND gate.

Supplementary Figure 2

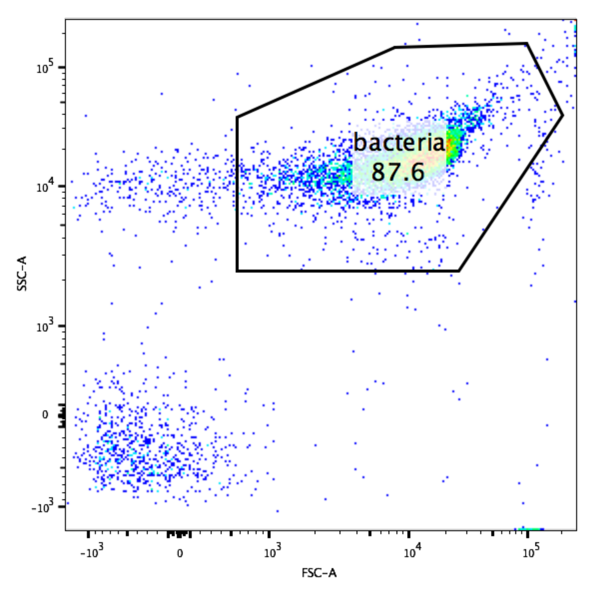

Supplementary Figure 2. Typical cytometry gating used.
